# Newly graduated nurses’ perceptions of their professional role and professional competency: a qualitative focus group study

**DOI:** 10.1186/s12909-023-04747-7

**Published:** 2023-10-11

**Authors:** Mirko Prosen, Sabina Ličen

**Affiliations:** Faculty of Health Sciences, Department of Nursing, University of Primorska, Polje 42, Izola, 6310 Slovenia

**Keywords:** Competence, Graduation, Nursing education, Retention, Strategies, Transition

## Abstract

**Background:**

In recent years, complex and rapidly changing healthcare settings have placed high demands on nursing graduates, who must effectively assume new professional roles with a wide variety of competencies. However, in an ever-altering environment it is impossible to teach students everything. This means that assessing nursing students’ perceptions of their ability to practise their competencies and assume their professional roles is critical for faculty to further develop the nursing education curriculum and to assist healthcare organisations in supporting the transition of graduates. The aim of the study was to explore newly graduated nurses’ perceptions of their new professional role and professional competency associated with this role.

**Methods:**

A qualitative study using the focus group method. The purposive sample included 18 nursing graduates with an average of 29 years, most of whom were female. The average time that had elapsed since graduation was 1.5 years. Four face-to-face focus group interviews were conducted. Thematic analysis was employed to identify themes and subthemes.

**Results:**

Three main themes describing newly graduated nurses’ perceptions were found: (1) significance of the transition period; (2) commitment to the nursing profession; and (3) perceived needs and challenges to professional competency.

**Conclusion:**

The transition from a safe academic environment to turbulent clinical practice is shaped by both graduates’ expectations and reality. The results of the study indicate a high level of commitment to the profession immediately after graduation and point to groups of competencies that need greater emphasis in the nursing curriculum. Although the responsibility for adequately preparing and supporting nursing students for their new professional roles rests with faculty and healthcare organisations, the responsibility for professional development should also lie on the students themselves.

## Introduction

Health systems have undergone significant and rapid changes in the last few years that have challenged not only the structure of the systems itself but also the professions that serve within them. Nurses, as one of the largest professional groups, are expected to take professional responsibility for continuously providing adequate care, protecting individuals’ lives and supporting daily living activities. To accomplish this, it is important to improve nursing competencies and utilise them in day-to-day nursing practice [1–3]. This is also important from the perspective of clearly defining parts of the nursing education curriculum that support the development of competencies and lead to nursing students’ successful transition to clinical practice [4].

Nursing competency and the concept underlying it has yet to be fully developed while the definition still lacks consensus. In general, nursing competency encompasses knowledge, skills, actions, values and attitudes that are all often interpreted through behavioural, generic and holistic approaches [1, 2, 5, 6]. Nursing competency also intertwines with professional characteristics of the profession like following ethical and moral standards, clinical reasoning abilities, communication skills and other elements that surround a nurse’s professional role [5]. The awareness of all these aspects of a nurse’s competency and scope of practice after graduation constitutes a key issue closely associated with professional standards, patient safety, credibility of the nursing profession [7] and, thus, the professionalisation of nursing.

In healthcare settings that are constantly changing, it is impossible to teach students everything [7], meaning that preparing nursing students for an adequate transition to clinical practice after they graduate demands effective strategies that ensure stability, consistency and support in assuming their new professional role. In this context, the evaluation of graduate nursing students’ perception of their ability to exercise their competency and assume their professional role is becoming crucial for faculty and healthcare organisations struggling with the shortage, turnover and absenteeism among nurses [3, 7, 8].

The aim of this study was to explore newly graduated nurses’ perceptions of their new professional role, their transition into this role, and the professional competency associated with it.

## Methods

### Research design

A qualitative descriptive design with a focus group discussion was adopted in order to obtain in-depth perceptions among new graduates about their transition into their new role as practicing nurses. This approach has been identified as important and appropriate for research questions focused on discovering the who, what, how and where of events or life experiences and gaining insights from informants regarding a poorly understood phenomenon. In addition, it enables flexibility in all phases of the research by providing rich, intertwined and comprehensive descriptions of various perspectives of the phenomenon under study by stimulating interaction within the group and the joint construction of meaning [9–11]. The main topic question guiding the focus group discussion was “How do you perceive your transition to your new role and your ability to exercise professional competency in everyday clinical practice?”.

### Setting

This study was conducted in Slovenia. The Slovenian healthcare system is a publicly-funded service organised on three levels of care. Hospital treatment is arranged on secondary and tertiary levels in general and specialised hospitals located around Slovenia, while primary care is organised on the local level, chiefly in community health care centres [12, 13]. Nurses as a professional group, like everywhere else in the world, account for the biggest group of healthcare professionals in the Slovenian health system. In 2019, there were 10.5 nurses per 1,000 inhabitants [14]. Undergraduate nursing education in Slovenia complies with the Directive on the Recognition of Professional Qualifications 2005/36/ES and 2013/55/EU as well as the guidelines of the Bologna Declaration [15]. Nursing in Slovenia is a regulated profession. Pursuant to the regulations in force, all nurses in Slovenia must be entered in the records and hold a valid licence to practise autonomously. The provision of nursing care encompasses planning, organization, leadership, nursing interventions, and their coordination and supervision in accordance with prevailing doctrine and ethical guidelines [16]. In 2019, after two decades of efforts by the national nursing and midwifery association, a law on “Professional competencies and activities of practitioners in nursing care” was approved by the Ministry of Health and entered into force, finally defining the formal and legal scope of the practice of nursing in Slovenia [17].

### Participants

We recruited participants through professional and social networks, as well as through snowball sampling. To be eligible for the study, participants had to be new graduates (Bachelor in Nursing), with no more than 2 years having passed since graduation, and working as practising nurses. All participants were full-time students during their studies and had no prior clinical experience; their sole clinical experience was acquired during their student clinical placements. The purposive sample included 18 participants divided into 4 focus groups.

### Data collection

The data were collected in January 2022 from four focus groups (two focus groups included four participants, and two focus groups included five participants). Previous research suggests that more than 80% of all themes are discoverable within two or three focus groups and 90% within three to six focus groups [10]. All focus groups were conducted face-to-face in a quiet conference room at the faculty. Participants had the possibility to choose a focus group based on their availability. The focus groups were moderated by one of the researchers who again explained the aim of the study and obtained written informed consent from the participants. Participants also completed a short, five-question long questionnaire on basic socio-demographic data (gender, age, time of graduation, years of experience as an RN, place of employment).

The focus group topic guide [18] consisted of structured questions based on components of the framework proposed by Nilsson, Engström, Florin, Gardulf and Carlsson [19] as it fitted the context and competencies of nurses in Slovenia [20]. During the interview, the moderator asked all the main questions set in the topic guide (Table 1) and, before moving on to the next question, gave participants an opportunity to add or discuss anything about the topic. The focus group interviews lasted approximately 1 h each. After the fourth focus group, new themes no longer emerged and the researchers decided to bring the data collection to a halt [11]. All focus group interviews were audio-recorded and transcribed verbatim by the researchers. No field notes were made during the interviews as the moderator wished to keep participants focused during the whole interview, although self-reflective field notes were made immediately after the focus group ended and subsequently aided in the data analysis phase.

**Table 1:** Focus group topic guide

Content structure	Examples
Beginning	How did you find yourself in your new role after graduating? Did you notice any difference between the image of nursing and nurses that you had during your studies and the real-life situation in clinical practice after you graduated?
Middle	How would you describe your current knowledge and skills in the field of nursing where you are employed? How would you describe your abilities to independently apply the nursing process? How would you describe your abilities related to accurate and effective documentation and administration of nursing care? How would you describe your abilities related to the leadership and organisation of nursing care, including supervising team members? How would you describe your abilities to teach, supervise and assess students? How would you describe your abilities to inform and educate patients and their relatives? How would you describe your abilities to translate knowledge into practice (evidence-based nursing)? How would you describe your abilities to assess patient health and prioritise their needs? How would you describe your abilities related with the use of ICT as support in nursing care? How would you describe your abilities and opportunities to respect patient autonomy, integrity and beliefs while delivering care? How would you describe your abilities to respectfully communicate with patients, relatives and staff? How would you describe your abilities about working with culturally diverse patients and team members? How would you describe your abilities related to pharmaceutical care? How would you describe your abilities in recognising unprofessional conduct and taking actions when/if it happens? How would you describe your abilities with respecting standards of care, guidelines and safety protocols in the workplace? How would you describe your ability to be continuously engaged with personal and professional development?
End	Anything else you would like to add?

### Data analysis

Before the analysis began, each transcript was cross-checked against the audio-recording for accuracy. Thematic analysis was used following the steps suggested by Bryman [11]: (1) re-read the transcripts to become thoroughly acquainted with the content; (2) generate codes; (3) merge these codes to create themes; (4) evaluate the higher-order of the codes/themes; (5) examine links and connections between the concepts and features of individual cases; and (6) write a compelling narrative and justify the theme conceptualisation. The data were analysed by both authors separately for each focus group interview using the NVivo 1.6.1. (QRS International Pty Ltd.) software.

### Rigor and trustworthiness

We followed the trustworthiness standards for qualitative research proposed by Lincoln and Guba [21]. To ensure the credibility, as transferability and dependability of the results, each step in the research process was documented in detail, as was the cultural context of the results. The audit trail was supported by the ‘research diary’ each researcher kept. Diary entries included were descriptions of the tasks performed, and were chronologically organised and self-reflective in nature. The two researchers worked on the analysis and together reached consensus on the final conceptualisation of the results, which contributed to their confirmation. In addition, the final results were discussed with colleagues outside the research team to learn about and understand the results from their perspective. During the analysis, comparing and contrasting data was conducted across cases as well as within individual cases. In presenting the findings, we selected meaningful and illustrative quotes that reflect the context of the theme, showcasing contrasts and relationships among concepts [22, 23].

### Ethical considerations

This study was conducted in line with the principles of the Declaration of Helsinki [24] and approved by the Ethical Committee of the Nurses and Midwives Association of Slovenia (No. 4-10-18). Participation was voluntary and anonymous. The researchers explained the purpose of the study and research methods used to the participants before the latter made a final decision to participate. Participants who agreed to participate signed an informed consent form and were told that they could withdraw from the study at any point, despite having given written consent. All data were treated confidentially pursuant to the European Union General Data Privacy Regulation [25].

## Results

### Participant characteristics

The 18 participants in the 4 focus groups were mostly female (n = 13). The participants’ age ranged from 24 to 50 years, with an average age of 29. Five participants had enrolled in the nursing programme as part-time students, while the rest were studying full time. The average time that had elapsed since graduating was 1.5 years, with the same number of years of work experience as an RN. Most participants had found employment at the primary (n = 7) and secondary (n = 7) levels and some at the tertiary (n = 2) level of healthcare after graduation. Two were employed in nursing homes.

### Graduates’ perception of their professional competence

**Fig. 1:**
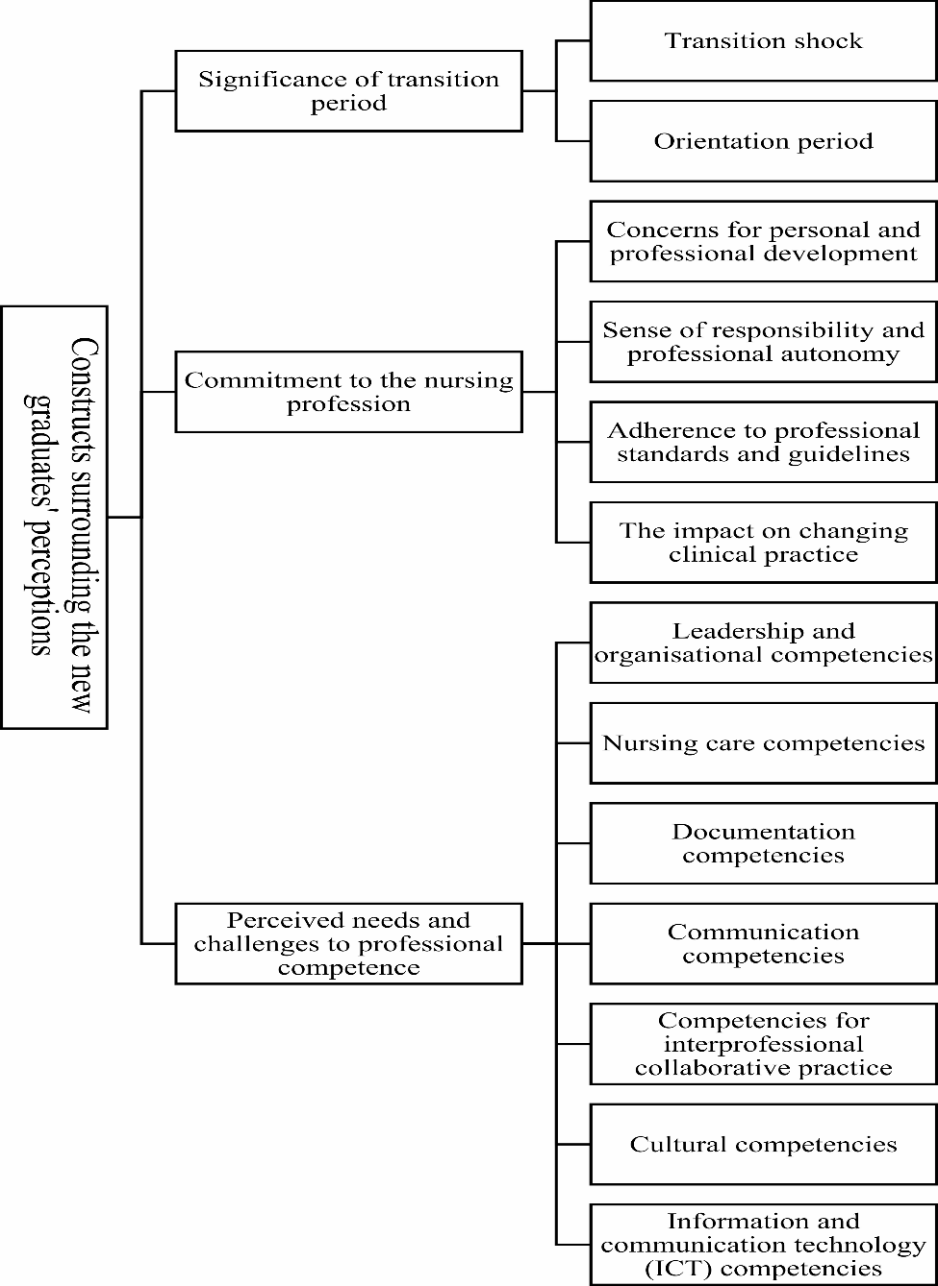
Identified themes and subthemes

Three themes emerged from analysis of the data: (1) the significance of the transition period; (2) commitment to the nursing profession; and (3) perceived needs and challenges concerning professional competency (Fig. 1).

#### Theme 1. Significance of the transition period

Some graduates who had started working in clinical practice were faced with a serious ‘reality check’ as they experienced what can clearly be defined as transition shock. The gap between their conception of the professional role of a nurse, the responsibilities that this role entails, and last but not least the true context of nursing as perceived during the study years compared to working in clinical practice was simply too intense for them to comprehend and adapt to.

*Every time it feels like ‘swimming’ and you try to adapt. I feel like the dreams of how smoothly things will go in practice are quickly shattered. In faculty, you are taught a kind of order, a systematic approach that gives you a sense of security but, once you are in practice, that sense of security is gone.*

*I’m ashamed to say this, but after I started working in clinical practice, I finally realised what nursing actually is. Clinical practice during studies doesn’t show you or prepare you enough for all the tasks you’ll have to do as a nurse. As a student, you just do not see things in the ‘background’, but when you are employed as a nurse you suddenly must deal with all kinds of tasks. You are shocked every day.*

This also explains why some felt that the orientation programme after graduation was too short and that the orientation period should be extended or in a few cases at least mentored. However, students who possessed additional clinical practice experience prior to graduating, either working full- or part-time in clinical settings during their studies, had experienced the transition to professional practice with much less intensity and stress. In addition, graduates’ perceptions of the orientation period varied from clinical setting to clinical setting, being more organised in some than in the others, demonstrating the lack of a systematic approach.

*Before I graduated, I worked part-time in the emergency room and since they knew I was going to graduate soon, they went out of their way to introduce me to the various competencies of a nurse.*

*During your studies, your idea of nursing is based on what you are told and read and, of course, to a smaller extent, what you see and practise. During this process, you are mentored, you are in control, and you know that someone is watching over you. But the reality hits you soon – all of that is over. The orientation period, in my opinion, should be longer and accompanied by a mentor.*

*There is a shortage of nurses everywhere, I understand that. I had a mentor, but on weekends I was on my own. I felt overwhelmed by responsibility. I realise that sometimes it’s good to be ‘thrown in the deep end and to swim’, but to be honest, I brought my work home and worried if I was doing everything right.*

#### Theme 2. Commitment to the nursing profession

Concerns about the personal and professional development of new graduates were prominent among most participants. Two of the greatest concerns related to the awareness of the importance of knowledge in practising one’s profession to meet high quality standards and the consequent need for lifelong learning as an essential part of this.

*I’m working in anaesthesia. I learned a lot at the faculty, don’t get me wrong, but I had to take up books in my hands again soon after graduating and learn and understand so many new things to properly function in the ward.*

*You have to have a broad horizon over things. You definitely must learn a lot more in addition to what you already know and this should be done continuously because things in our clinical practice are rapidly changing.*

Many of the graduates had noticed that “continuously seeking new information and knowledge” is perhaps more associated with younger generations of nurses, rather than nurses with a longer tenure.

*I have the feeling that younger generations of nurses more intensely seek new information in comparison to nurses with a longer tenure. I’m not sure why that is. Perhaps it is lower job satisfaction, I don’t know. I think I was educated in a system that pushed us forward and created a habit of seeking new knowledge. I personally try to keep track of new things in the field. From a personal perspective, I think I am also evolving as a person, becoming a better person.*

A sense of responsibility and professional autonomy are strongly present in most graduates at this stage of their career. Responsibility was also often accompanied by openly expressed feelings of anxiety and burden arsing from the new role. However, a clear line was drawn in defining the nursing scope of practice and boundaries that distinguished their own competencies from those of other professions.

*I must admit that I was quite anxious at the beginning. As a student, you do not have a sense of responsibility, and then suddenly you start working as a nurse and realise that you are fully responsible for everything you do. The mistakes you make are yours, no one else’s.*

*After graduating, you feel so relieved because it took you so long to get here, but that relief soon ends because soon there are new responsibilities that come your way that you were not even aware of.*

*Concerning nursing competencies, I’m well aware what falls within my scope of practice and what within medicine or any other profession. There are times when patients do not understand this, and demand things which are not within my jurisdiction.*

Professional commitment is also demonstrated by the graduates’ adherence to professional standards, protocols and policies, reflecting responsible behaviour in their immediate nursing care and making them sensitive to the professional misconduct of others. Still, because they are early in their careers, they do not always have sufficient internal strength and authority to speak up.

*It is important that we adhere to standards of care and protocols that guide our work, but practice also shows a different picture. For example, consideration for the patient’s privacy in a hospital room with several patients is rarely in focus while providing care.*

*You notice certain cases of professional misconduct among colleagues, but I do not say anything because they have a senior position and I have just started working and I do not think that I can afford to criticise.*

Another important aspect of professional commitment that the majority of graduates pointed out was the idea of advancing nursing practice and infusing it with innovative and evidence-based approaches. Among some graduates, doubts had appeared about the current working procedures and process, yet they were well aware of the “appropriate timing”.

*Sure, you can change the nursing practice, but not immediately after graduation, at least not as effectively. I speak for myself, but the faculty encouraged us to take an evidence-based approach, and if I have doubts about the things I am doing, I must address those doubts.*

#### Theme 3. Perceived needs and challenges to professional competency

Leadership and organisational competencies were recognised as one of the most significant competencies among graduates. This role was associated with both leadership and management tasks and presented a challenge especially because it was regarded as stressful and some did not feel confident in this role.

*I motivated the team members and delegated the assignments. They had a job to be done. I knew what I had to do and did it.*

*It was difficult for me. I do not feel that I’m the right person to order people around and tell them what to do.*

During the interviews, the graduates raised the topic of interprofessional collaborative practice and related competencies, acknowledging that the quality and safety of care within the health system depends on the partnership and collaboration of different professions. They felt this collaboration was positive and something that should be encouraged during their studies.

*We had a dialysis patient […] with multiple health and nursing problems. Doctors, pharmacists and nurses worked together to solve her problems. It was great to see how teamwork works and that optimal care is not just the result of one healthcare professional group.*

Reflections on the graduates’ nursing care competencies revealed a gap between expectations and the reality of clinical practice. While they believed (and had been educated to believe) that nursing should focused on the patient, in reality this was not the case. The nursing process, as it was thought of at the faculty, was rarely practised in this manner in the wards.

*During my studies, we were much more focused on the patient, but in clinical practice, time with the patients is limited and you just lose the ‘connection’ with them.*

*In the nursing home where I work, the nursing process does not work the way I learned it. I mean NANDA nursing diagnoses etc. I work on the same principle that the nursing process is based on, but because of the nursing shortage, documentation, planning and other elements are put to one side.*

This statement also conceals another aspect that was noted more broadly in the focus groups, e.g., documentation competencies that could be considered to form part of nursing administration. In this context, ICT (Information and Communication Technology) competencies are found valuable as most of this process is done digitally. While the graduates were aware of the importance of documenting nursing care, they felt overwhelmed by bureaucracy behind their work.

*Documenting what was done or not done is very important, for patients and for myself. I just sometimes feel that I am working more with documents than with patients.*

The graduates were also aware that communication competencies are skills that develop over time. At this stage of their work, it seems very important that they receive appropriate feedback, especially from patients.

*Communication is a skill that develops over time. I think it’s a skill that developed quite quickly after graduating, but I realise that it depends on the person. […]. For me, it’s important to get feedback from patients.*

Communication skills were also associated with cultural competencies. A few graduates described cases of caring for patients from different cultures and their awkwardness in overcoming the language barrier. Most felt they were insufficiently prepared to deal with the cultural issues of nursing care.

*I have a lot of patients coming from Albania and the language barrier is impossible to overcome. I had to educate myself a lot regarding their culture and habits. If I could, I would change the curriculum. They (the faculty) should put greater emphasis on this aspect of care.*

## Discussion

Exploring the experiences and perceptions of new graduate nurses concerning their professional roles and competencies helps to understand their transition from the faculty to clinical practice and the challenges they encounter while practising their profession. The new insights that emerged may contribute to the future planning of policies and strategies by academia, professional associations, and healthcare organisations. The findings of this study indicate that the graduates tied their perceptions to three main themes, e.g., transition and support during the transition, a sense of commitment to the profession, and challenges related to the scope of the practice of nursing.

The transition from the role of nursing student to professional nurse is a challenging and multifaceted process. The importance of a newly graduated nurse’s successful transition to professional practice has been associated with greater job satisfaction and a lower likelihood of leaving the profession. The transition from the safe academic environment to the turbulent clinical practice is shaped both by graduates’ expectations and the reality of the situation [4]. Newly graduated nurses are expected to take on different roles and effectively care for multiple patients, each bringing their own challenges and complexities, in order to succeed in the role of a professional nurse [8, 26]. The study results indicate that graduates, particularly those with less clinical experience before graduation, faced what has been labelled a “transition shock”, reflecting the emotional, physical, sociocultural and intellectual experiences of graduates as they transitioned [27, 28]. Feelings of insecurity and anxiety led the graduates in this study to perceive the orientation programme as too short and lacking appropriate mentorship/preceptorship. Indeed, the literature states that the most stressful and challenging time for new nursing graduates is their first few months of clinical practice when they transition from a student role to one of practising nurse [29, 30]. In addition, the results show the approach of the orientation programme varies from institution to institution, which means that there is no standard way for preparing graduates for their new roles.

These transition-related aspects should be considered while developing and ensuring appropriate support mechanisms during this time to overcome barriers arising from the transition shock or other work environment influences. Fostering the ‘resilience’ of graduates, which may be defined as a nurse’s psychological trait, ability and strength to withstand, overcome and grow from adversity, leads to a successful transition [29]. Preparing the next generation of graduates to successfully assume tasks in the complex environment of healthcare institutions, where the circumstances change rapidly and the health status of patients is often deteriorating, requires innovative and transformative approaches to education. On the other hand, one can also find healthcare institutions that must work in partnership with academia to provide an environment that is supportive of newly graduated nurses and ensures the conditions for the development of their resilience and professional identity [8, 26, 30].

Traits of professional commitment were strongly present among the newly graduated nurses. A sense of responsibility, respect for the jurisdiction of one’s profession and adherence to professional standards and policies were also noticeable among the graduates. Although the concern for personal and professional development among the new graduates is not surprising, it is reassuring that it is present to this extent so early in their career, which is obviously a consequence of successful secondary socialisation. This process has long been recognised as an important vehicle for conveying norms and values associated with enhancing social capital and thereby building an empowered professional identity [31]. Nursing educators and academia must continue to implement and evaluate ways of strengthening the professional identity of nursing students. An important approach is to establish learning environments that incorporate inspirational leadership, effective management, and the creation of a positive partnership. Nursing programmes must also ensure that members of clinical nursing faculty are strong role models for nursing students [30] and in this role should familiarise students with their future professional role and competencies in a real-world context. This would contribute positively to the transition from student to practising nurse and reduce the anxiety that some graduates experienced with their new role.

Nursing students are today expected to possess adequate competencies upon graduation [6], yet the question remains of how they perceive their level of competency and which competencies does clinical practice require of them. Contemporary nursing practice requires complex decision-making skills in a rapidly changing clinical environment and is simultaneously a social practice that intertwines autonomy, interprofessional collaboration and other elements associated with the professionalisation process. New graduate nurses must be prepared to practise safely, accurately and compassionately [8, 26, 30]. The results of this study show that nursing administration competencies, including leadership and organisational competencies, documentation competencies, communication and ICT competencies, competencies for interprofessional collaborative practice and cultural competencies, are among the most important and, at the same time, challenging competencies as perceived by newly graduated nurses. These are also professional competencies that take time to evolve. Adapting to the new role and acquiring of these competencies was initially associated with a lack of self-confidence for many graduates and therefore represented a stressful situation, as found in previous studies [30]. Healthcare institutions should be more sensitive to these experiences while designing their orientation programmes since it is not only important for them and the new graduates to develop these competencies, but also for the patients.

Based on these findings, future research could delve deeper into the transition of students to practice and explore the concept of resilience among newly graduated nurses after clinical immersion in the healthcare environment. It could also explore the perceptions of nursing educators and clinical mentors as well as the expectations of healthcare institutions as regards the role of newly graduated nurses. Exploring these issues is particularly important in the light of nursing shortages and turnover. From the perspective of professional competencies and professionalisation, the regular assessment of the level of competency achieved by nursing students and new graduates would benefit the nursing curriculum and orientation/preceptorship programme designers and should involve a combination of both research paradigms.

This study has some limitations. First, the majority of students were graduates of a single faculty. Including a more diverse distribution of graduates from other faculties and clinical settings would introduce the comparative perspective. Second, the cultural context of the study must be considered because it could limit the transferability of the results. Third, despite the interactive and dynamic nature of the focus group being positive, it might also be a potential limitation by inadvertently influencing the responses of participants reluctant to express their opinions. The moderators attempted to prevent this by using a variety of moderating techniques.

## Conclusion

This study provides insight into the experiences and perceptions of newly graduated nurses regarding their professional roles and the scope of their competences at this stage of their career. Successful transition from one role to another is the responsibility of educational and healthcare institutions and professional bodies, not only for newly graduated nurses but also for patients and the delivery of compassionate, safe and high-quality nursing care.

## Data Availability

The anonymised data are available from the corresponding author upon reasonable request as the participants were assured that it would remain confidential.
